# sEMG Activity in Superimposed Vibration on Suspended Supine Bridge and Hamstring Curl

**DOI:** 10.3389/fphys.2021.712471

**Published:** 2021-08-11

**Authors:** Joan Aguilera-Castells, Bernat Buscà, Jordi Arboix-Alió, Adrià Miró, Azahara Fort-Vanmeerhaeghe, Javier Peña

**Affiliations:** ^1^Faculty of Psychology, Education Sciences, and Sport Blanquerna, Ramon Llull University, Barcelona, Spain; ^2^School of Health Science Blanquerna, Ramon Llull University, Barcelona, Spain; ^3^Sport and Physical Activity Studies Centre (CEEAF), University of Vic-Central University of Catalonia, Vic, Spain; ^4^Sport Performance Analysis Research Group (SPARG), University of Vic-Central University of Catalonia, Vic, Spain

**Keywords:** instability, vibration, lower limb, suspension training, electromyography

## Abstract

Traditionally in strength and conditioning environments, vibration has been transmitted using platforms, barbells, dumbbells, or cables but not suspension devices. This study aimed to examine the effects on the lower limb of applying superimposed vibration on a suspension device. Twenty-one physically active men and women performed supine bridge and hamstring curl exercises in three suspended conditions (non-vibration, vibration at 25 Hz, and vibration at 40 Hz). In each exercise condition, the perceived exertion scale for resistance exercise (OMNI-Res) was registered, and the electromyographic signal was assessed for gastrocnemius (medialis and lateralis), biceps femoris, semitendinosus, gluteus maximus, and rectus femoris. A linear mixed model indicated a significant fixed effect for vibration at 25 Hz and 40 Hz on muscle activity in suspended supine bridge (*p* < 0.05), but no effect for suspended hamstring curl (*p* > 0.05). Likewise, the Friedman test showed a significant main effect for vibration at 25 Hz and 40 Hz in suspended supine bridge (*p* < 0.05) but not for suspended hamstring curl (*p* > 0.05) on OMNI-Res. *Post hoc* analysis for suspended supine bridge with vibration at 25 Hz showed a significant activation increase in gastrocnemius lateralis (*p* = 0.008), gastrocnemius medialis (*p* = 0.000), semitendinosus (*p* = 0.003) activity, and for semitendinosus under 40 Hz condition (*p* = 0.001) compared to the non-vibration condition. Furthermore, OMNI-Res was significantly higher for the suspended supine bridge at 25 Hz (*p* = 0.003) and 40 Hz (*p* = 0.000) than for the non-vibration condition. Superimposed vibration at 25 Hz elicits a higher neuromuscular response during the suspended supine bridge, and the increase in vibration frequency also raises the OMNI-Res value.

## Introduction

Nowadays, strength and conditioning practices combine resistance exercises and other training methods such as eccentric overloads, unstable surfaces, and suspension devices for improving strength and power performance (Maté-Muñoz et al., 2014; Behm et al., 2015; Suchomel et al., 2019). Similarly, coaches and fitness enthusiasts have also used mechanical vibrations as an alternative or complement to strength and explosive training (Hammer et al., 2018). The effects of vibration training have been widely studied on neuromuscular performance (Alam et al., 2018), flexibility (Fowler et al., 2019), and balance control (Ritzmann et al., 2014; Sierra-Guzmán et al., 2018). This method transfers the vibratory stimulus on the muscle belly and tendon directly (local) or indirectly (e.g., vibrating platforms) to elicit the tonic vibration reflex (Cardinale and Bosco, 2003). Platforms are the most commonly used piece of equipment in sports training to transfer whole-body vibration (WBV) and modify the stimulus through the type of vibration (side-alternating vibration or synchronous vibration), frequency (in Hz), amplitude (peak to peak amplitude), position, and time of exposure (Cardinale and Wakeling, 2005; Issurin, 2005).

WBV has been combined with different training methods, and lower-body resistance exercises (bodyweight or extra loads) performed under static and dynamic conditions (Rittweger, 2010). Several studies have shown the positive effects of performing WBV squats or other exercises such as lunges or Bulgarian squats on muscle strength and jump ability (Rehn et al., 2006; Fort et al., 2012; Osawa et al., 2013). However, the effect of vibration training on dynamic exercises with heavy loads (squats) did not improve maximal strength and jump performance using WBV at 40 Hz (Rønnestad, 2004) or 50 Hz at < 1 mm of amplitude (Hammer et al., 2018). Contrarily, dynamic squat training (6 sets of 6 reps; with an individual optimal load) performed on a vibration platform (30 Hz at 4 mm of amplitude) combined with repeated sprint training (3 sets of 6 reps of 20 meters shuttle run with 180° change of direction) (Suarez-Arrones et al., 2014) or functional eccentric-overload exercises (8 exercises between 6 to 10 reps with an inertial load ranged from 0.27 Kg⋅m^–2^ to 0.11 Kg⋅m^–2^) (Tous-Fajardo et al., 2016) elicited higher performance than traditional resistance training (lunges, half-squats, and calf raises; 50–100% body mass) on sprint, change of direction, and jumping performance. Furthermore, blood flow restriction training combined with WBV resistance training (30 Hz and parallel squat with dynamic loading) improved critical power, overall capillary-to-fiber ratio, and total lean body mass in endurance-trained men (Mueller et al., 2014). Considering acute effects, Bush et al. (2015) reported a post-activation potentiation effect on knee extension torque after exposing healthy participants to a WBV dynamic squat with bodyweight resistance (30 Hz and 4 mm of amplitude). Additionally, Aguilera-Castells et al. (2019) showed that combined WBV (40 Hz) with a suspended device elicited higher muscle activity than the suspended condition for hip and thigh muscles in the dynamic lunge bodyweight resistance.

In the studies mentioned above, the WBV was provided with a vibration platform to assess the effects of combining vibration and resistance training on different neuromuscular performance variables such as maximal strength, mechanical power, jumping ability, or muscle activity. However, to transfer the vibratory stimulus to the upper body, several devices with superimposed vibration have been used in the past, such as dumbbells (Bosco et al., 1999; Cochrane and Hawke, 2007), bars (Poston et al., 2007; Mischi and Cardinale, 2009; Moras et al., 2010; Xu et al., 2013), and cables (Issurin and Tenenbaum, 1999; Issurin, 2010). Likewise, superimposed vibration has been used to study the training effects on the lower body. Thus, the addition of vibration (30 Hz at 2.5 mm of amplitude) had no effects during four weeks of dynamic calf-raise on a seated rig (75–90% 1RM) (Carson et al., 2010). However, superimposed vibration on a BOSU (35–40 Hz and 2 to 4 mm of amplitude) enhanced the reaction time of peroneus brevis, longus, and tibialis anterior in athletes with chronic ankle instability during six weeks of training (Sierra-Guzmán et al., 2017). Furthermore, surface electromyography (sEMG) has been used to evaluate the activity of different muscles during an exercise with superimposed vibration (Xu et al., 2015). Thus, Marín and Hazell (2014) found higher activation of the gastrocnemius medialis, vastus medialis, and multifidus during 60° knee flexion static half-squats with superimposed vibration on a BOSU (30 Hz and 50 Hz and 1 mm of amplitude) in comparison to the stable condition. To the best of our knowledge, there are only four devices with superimposed vibration allowing the lower body training. Two of these devices are similar to vibration platforms, consisting of a small platform to improve flexibility in gymnasts (Sands et al., 2006; Kinser et al., 2008) and a platform with a bi-engine that provides vibration on a leg press machine (Pujari et al., 2019). The other two devices are Vibrosphere (ProMedvi), a superimposed vibration wobble board (Cloak et al., 2013), and Vibalance (Viequipment), a platform that combines vibration with different degrees of instability even though neither of these devices superimposed vibration on suspension straps.

Although the squat and its variations are the most used resistance exercises in WBV, the most demanded actions in team sports are sprinting, jumping, and cutting, generating numerous lateral actions and unilateral movements that demand horizontal force production (Gonzalo-Skok et al., 2017). Hence, the use of functional equipment such as suspension straps allowing exercises in multiple planes (Bettendorf, 2010), the inclusion of exercises based on the force-vector theory such as the barbell hip thrust to improve horizontal force production (Loturco et al., 2018; Neto et al., 2019), and preventive training on the hamstrings muscle complex (Rey et al., 2017; Bourne et al., 2018a) are commonly used in strength and conditioning team-sport programs. In the last decade, injuries to the hamstrings complex have increased in different team sports, especially in soccer, with an injury rate ranging between 15 and 50% (Al Attar et al., 2017). To strengthen the hamstrings complex (biceps femoris, semitendinosus, and semimembranosus), different bilateral and unilateral exercises, such as the deadlift, supine bridge, leg curl, glute-ham raise, or Nordic Hamstring have been included in injury prevention programs (Bourne et al., 2017). Thus, the suspended supine bridge and the hamstring curl were selected in the current study because of their popularity in hamstrings preventive programs (Malliaropoulos et al., 2012; Youdas et al., 2015). On the one hand, the supine bridge is a bodyweight exercise demanding the posterior hip and thigh muscles as gluteus maximus and hamstrings (Jang et al., 2013; Kim and Park, 2016; Lehecka et al., 2017; Marín and Cochrane, 2021), and it is a recommended exercise for strengthening and prevent injuries in hamstrings and lower back muscles (Ekstrom et al., 2007). This exercise is considered a variation of the hip thrust, where back and feet are placed on the ground, thus increasing the difficulty by modifying the position of the feet on a bench or an unstable surface (i.e., suspension device) (Tobey and Mike, 2018). Conversely, the hamstring curl is considered an open kinetic chain knee dominant exercise (Malliaropoulos et al., 2015) that uses body weight as resistance and aims to develop the strength and endurance of the hamstring muscles (Dawes, 2017).

Accordingly, a vibratory system for suspension training has been designed to provide an indirect and superimposed vibration on the suspension device, allowing a wide range of exercises in different planes. Therefore, the main objective of the present study was to examine the effects of the vibration device on muscle activation in the dynamic suspended supine bridge and hamstring curl exercises. It was hypothesized that the superimposed vibration on the suspension device would obtain a superior muscle activation than the suspended condition without vibration in both exercises. Additionally, it was also hypothesized that the OMNI-Res perceived exertion scale for resistance exercise would be higher in the suspended condition with vibration than the condition without vibration in each of the two exercises.

## Materials and Methods

### Participants

Twenty-one physically active participants males (*n* = 15, mean age = 23.3 ± 2.8 years, height = 1.8 ± 0.0 m, body mass = 77.8 ± 6.9 kg, body mass index = 24.1 ± 1.8 kg⋅m^–2^, suspension training experience = 4.2 ± 1.5 years) and females (*n* = 6, mean age = 22.6 ± 1.0 years, height = 1.6 ± 0.0 m, body mass = 56.6 ± 2.9 kg, body mass index = 21.5 ± 1.7 kg⋅m^–2^, suspension training experience = 3.8 ± 1.9 years) were voluntarily recruited to take part in the study. Participants experienced in suspension training for less than one year, not performing 30 min of physical activity at least three times a week, or having pain or injury related to cardiovascular, musculoskeletal, or neurological diseases were excluded from the study. Additionally, before the familiarization session, an informed consent form was provided and signed by all participants after receiving a detailed explanation, both in verbal and written form, of the experimental procedures, benefits, and risks of participating in the study. They also answered the Physical Activity Readiness Questionnaire (PAR-Q) to determine potential health risks associated with physical exercise (Warburton et al., 2011). Before the familiarization and test session, all participants were asked to refrain from high-intensity physical activity 24 h before the test session and avoid drinking, eating, or consuming stimulant substances (e.g., caffeine) 3–4 h before the test session. This study was approved by the Ethics and Research Committee Board in the Blanquerna Faculty of Psychology and Educational and Sport Sciences at Ramon Llull University in Barcelona, Spain, with reference number 1819005D. The requirements specified in the Declaration of Helsinki (revised in Fortaleza, Brazil, 2013) were complied with and implemented in all study protocols.

### Experimental Design

A cross-sectional study design was carried out to determine the effect of a vibratory system for suspension training on muscle activation in different lower limb muscles. Participants performed supine bridge and hamstring curl exercises in three suspension conditions: (a) non-vibration, (b) vibration at 25 Hz, and (c) vibration at 40 Hz. In all the above-mentioned conditions, muscle activation of the rectus femoris, biceps femoris, semitendinosus, gluteus maximus, gastrocnemius medialis, and lateralis was assessed and compared using sEMG. Muscle activation was normalized and expressed as a percentage of maximum voluntary isometric contraction (% MVIC). In addition, the OMNI-Perceived Exertion Scale for Resistance Exercise (OMNI-Res) was recorded to compare perceived exertion in each exercise condition.

### Procedures

**FIGURE 1:**
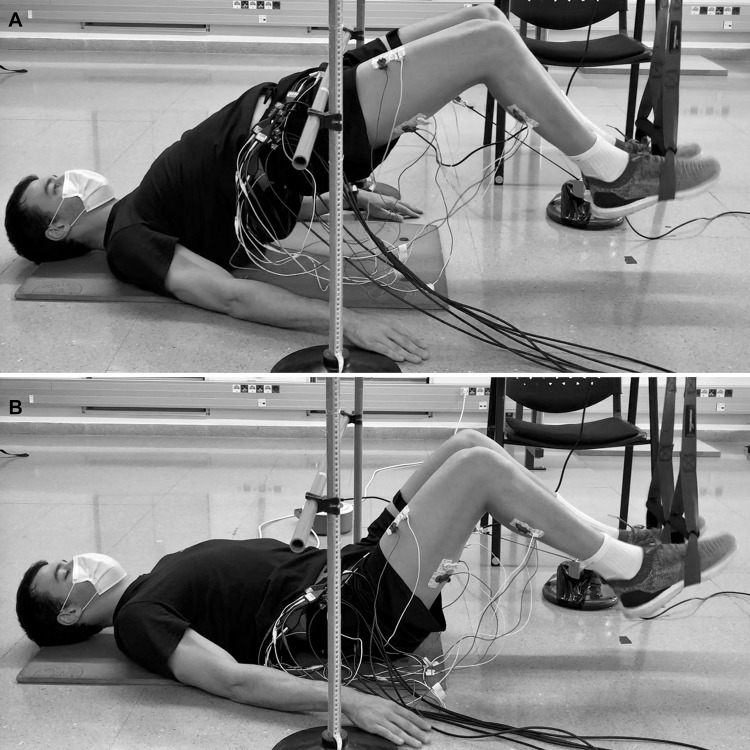
Suspended supine bridge: upper **(A)** and lower **(B)** position.

**FIGURE 2:**
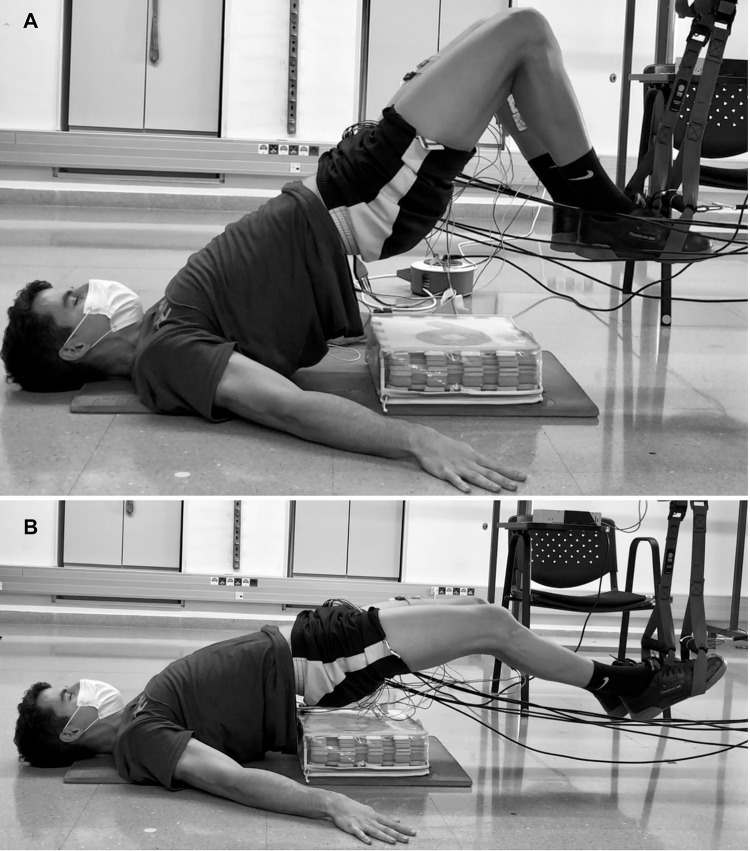
Suspended hamstring curl: upper **(A)** and lower **(B)** position.

A familiarization session was conducted one week in advance of the test session. In this session, participants performed two sets of five repetitions of each supine bridge and hamstring curl under suspended conditions (non-vibration, vibration at 25 Hz and 40 Hz), and the researchers collected anthropometric data such as age, height, and weight. The test session took place one week later in the morning at the same time as the familiarization session. The test session began with a standardized warm-up consisting of 10 min of cycle ergometer while maintaining a cadence of 100 W at 60 revolutions per minute, two sets of eight repetitions of a unilateral stiff-leg deadlift, two sets of five repetitions of Nordic hamstring assisted with an elastic band, and two sets of eight repetitions of unilateral straight knee bridge. Next, surface electrodes were placed on the dominant lower limb (Criswell and Cram, 2011), which was established subjectively by asking participants which leg they would use to kick a soccer ball (Meylan et al., 2009). Before performing the different supine bridge and hamstring curl conditions, maximal voluntary isometric contraction (MVIC) tests were performed on the rectus femoris, biceps femoris, semitendinosus, gluteus maximus, gastrocnemius medialis, and lateralis in order to obtain a baseline value and normalize the electromyographic signal (Halaki and Ginn, 2012). Afterward, participants performed the different supine bridge and hamstring curl conditions in a randomized order. For the suspended supine bridge exercise, the distance between the crista iliac and the cradle of the suspension device was standardized as 75% of the leg length, and the hip elevation was controlled with customized stoppers (similar to hurdles), starting the exercise with the lower back, arms, and hands in contact with the ground (Figure 1). For the suspended hamstring curl, the distance between the crista iliac and the device’s cradles was also 75% of the leg length, and the starting position of the exercise was standardized by laying the lower back and gluteus on a foam surface with a height corresponding to 20% of the leg length. Participants were instructed to begin with a complete knee extension in this exercise, release the lower back and gluteus on the foam surface, keep their arms and hands flat on the floor, perform a knee flexion, and then return to the starting position (Figure 2). The participants were instructed to place their feet inside the suspension device cradles with plantar flexion and to hold this position during all the repetitions in both exercises.

From each dynamic condition of the exercise, participants performed five repetitions with a two-minute rest between attempts. The pace of each repetition was controlled with a metronome giving a rate of 60 beats per minute, and the range of movement was controlled with a positional encoder (WSB 16k-200; ASM Inc., Moosinning, DEU) by attaching the tether to the thigh or the cradle of the suspension device in the supine bridge and the hamstring curl, respectively.

The movement signal recorded by the positional encoder in each repetition of the exercises was used to determine the concentric and eccentric phases of the movement. The positional encoder signal was divided in two for each repetition, establishing that the concentric phase or the ascent phase for the suspended supine bridge ranged from the initial position to the maximum hip extension (highest position) and for the suspended hamstring curl from the initial position to the knee flexion (highest position). In both exercises, the eccentric phase ranged from the highest position to the initial position (lowest position). The positional encoder determined the beginning and the end of each repetition, thus establishing the range of motion in the same acquisition timeline of the BIOPAC MP-150 system (BIOPAC System, Inc., Goleta, CA, United States) sEMG signal. Those attempts that did not follow the proper technical execution indicated by the researchers were discarded and repeated, providing the two-minute rest between trials. A TRX Suspension Trainer (Fitness Anywhere, San Francisco, CA, United States) was used for both exercises, with the device anchored to the ceiling. The distance between the floor and the suspension device cradles was standardized as 30% of the leg length of each participant. A vibratory suspension training system was used under vibration conditions (25 Hz and 40 Hz) and fixed between the ceiling anchor point and the suspension device. The vibratory system provided vibration to the suspension device by converting the rotary motion of an electric motor into a vertical motion, which caused the displacement of a connecting rod with an amplitude of 8 mm (peak to peak), and the motor rotation frequency was regulated with a potentiometer.

### Electromyography

The recording and analysis of sEMG of each muscle during each repetition under the suspended supine bridge and hamstring curl conditions (non-vibration, vibration at 25 Hz and 40 Hz) was performed with a six-channel BIOPAC MP-150 (sampling rate: 1.0 kHz) and AcqKnowledge 4.2 software (BIOPAC System, Inc., Goleta, CA, United States). Before placing the electrodes (Biopac EL504 disposable Ag-AgCl) over the rectus femoris, biceps femoris, semitendinosus, gluteus maximus, gastrocnemius medialis, and lateralis from the dominant leg, the skin area of the participants was prepared by shaving, exfoliating, and cleaning with alcohol to reduce impedance from dead surface tissues and oils. Following SENIAM recommendations (Hermens et al., 2000), the rectus femoris electrodes were placed at half the distance between the anterior superior iliac spine and the superior part of the patella; for the biceps femoris and semitendinosus at half the distance between the ischial tuberosity and the lateral epicondyle (biceps femoris) or medial epicondyle (semitendinosus) of the tibia; the gluteus maximus at half the distance from the sacral vertebrae and the greater trochanter; for the gastrocnemius medialis over the most prominent bulge of the muscle, and in the gastrocnemius lateralis at 1/3 of the distance between the head of the fibula and the heel. All electrodes were placed at an inter-electrode distance of 2 cm and were oriented longitudinally to the direction of the muscle fibers. In addition, a reference electrode was placed on the crista iliac. The sEMG signal was bandpass filtered at 10–500 Hz using a 4th order 50 Hz Butterworth notch filter, and the root mean square (RMS) was calculated. In order to normalize the results of muscle activation of each of the muscles analyzed, MVIC tests were performed on the dominant leg with three MVIC of five seconds, recruiting gradually up to the maximum for two seconds and maintaining the MVIC for three seconds, with a three-minute rest between MVIC following Jakobsen et al.’s (2013) procedures. The position of each muscle used to achieve the MVIC was based on Konrad’s (2006) protocol. Thus, the MVIC for the rectus femoris consisted of 90° seated single-leg knee extension; the MVIC for the biceps femoris and semitendinosus of 20–30° prone-lying single-leg knee flexion; the MVIC for the gluteus maximus in a supine-lying single hip extension; and the MVIC for the gastrocnemius medialis and lateralis in 90° seated ankle plantar flexion. All MVIC tests were against an immovable resistance; for the rectus femoris, biceps femoris, semitendinosus, and gluteus maximus, an ankle brace was used that was attached to a cable anchored to a stretcher. For the gastrocnemius medialis and lateralis, a horizontal leg press machine was used. The MVIC values obtained in each muscle mentioned above were used to normalize the RMS signal and report the muscle activation as % MVIC. For each exercise condition, the peak sEMG of each studied muscle during the concentric (ascending trajectory), and eccentric (descending trajectory) phase was analyzed, excluding the first and fifth repetition from the data analysis. Additionally, muscle activation levels recorded under the supine bridge and hamstring curl conditions were categorized as very high (> 60% MVIC), high (41–60% MVIC, moderate (21–40% MVIC), and low (< 21% MVIC) (Escamilla et al., 2010).

### OMNI-Perceived Exertion Scale for Resistance Exercise

This scale was used to register the perceived subjective exertion experienced during the suspended supine bridge and hamstring curl conditions (non-vibration, vibration at 25 Hz and 40 Hz). Once participants completed an exercise condition, they were asked to assess their perception of exertion. Participants were instructed during the familiarization session to follow the instructions for the OMNI-Res assessment by Robertson et al. (2003). During the familiarization and test session, a visual OMNI-Res scale was used, through which participants indicated the value of perceived exertion on a range from 0 to 10, where 0 indicated an extremely easy exertion (perception lower than that experienced during an unweighted repetition) and 10 an extremely hard exertion (perception higher than that experienced lifting 1 RM). The OMNI-Res values for each exercise condition were analyzed as mean OMNI-Res.

### Statistical Analysis

Statistical data analyses were carried out using the SPSS statistical package version 26 (SPSS Inc., Chicago, IL, United States). G^∗^Power (version 3.1.9.6; University of Dusseldorf, Dusseldorf, Germany) was used to calculate the sample size with power analysis and determined an effect size 0.29 SD with an α level of 0.05 and power at 0.95. All dependent variables showed a normal distribution, confirmed with the Shapiro-Wilk test, and met the inferential parametric assumptions, except the OMNI-Res. The global activity variable was calculated as the global mean of the six analyzed muscles. The effect of exercise condition on muscle activation (rectus femoris, biceps femoris, semitendinosus, gluteus maximus, gastrocnemius medialis and lateralis, and global activity) was assessed using a linear mixed model analysis considering the activation of each muscle as the dependent variable, the exercise condition as the fixed effect and the participants as a random effect. In case of a significant fixed effect, *post hoc* comparisons were made. Moreover, a non-parametric Friedman test was carried out to determine the effect of exercise conditions on the OMNI-Res. For significant main effects, a *post hoc* Wilcoxon test analysis with Bonferroni correction was applied. For pairwise comparison, Cohen’s *d* effect size (Cohen, 1988) and 90% confidence intervals (CI) were also calculated. Effect size was interpreted as trivial (*d* < 0.2), small (*d* ranging from 0.2 to 0.6), moderate (*d* ranging from 0.6 to 1.2), large (*d* ranging from 1.2 to 2.0), and very large (*d* > 2.0) (Hopkins et al., 2009). Statistical significance was set at *p* < 0.05, and all data were expressed as mean ± standard error of the mean (SE).

## Results

The sEMG activity of each muscle and the global activity during the concentric and eccentric phase of the suspended supine bridge and the suspended hamstring curl under non-vibration, vibration at 25 Hz, and 40 Hz conditions are shown in Tables 1, 2, respectively. Moreover, for the percentage of change of the analyzed muscles in the different suspended supine bridge and hamstring curl conditions, see Supplementary Tables 1, 2, respectively.

**TABLE 1:** The sEMG activity for each analyzed muscle under suspended supine bridge conditions.

		Suspended supine bridge				
Exercise phase	Muscle group	Non-Vibration	Vibration at 25 Hz	Vibration at 40 Hz		
		Mean ± SE	Mean ± SE	Mean ± SE	F	*p*
Concentric	Rectus femoris	1.7 ± 0.3	1.8 ± 0.4	2.0 ± 0.5	0.20	0.815
	Biceps femoris	19.1 ± 1.6	20.2 ± 1.6	19.6 ± 1.8	0.72	0.490
	Semitendinosus	19.7 ± 1.4	22.9 ± 1.5^a^	23.2 ± 1.7^a^	9.05	0.001
	Gluteus maximus	14.8 ± 1.7	16.1 ± 2.3	16.6 ± 2.2	1.79	0.178
	Gastrocnemius medialis	30.2 ± 2.0	37.4 ± 2.1^ab^	32.8 ± 1.8	9.71	0.000
	Gastrocnemius lateralis	36.5 ± 3.1	41.7 ± 3.1^a^	38.6 ± 3.1	5.19	0.010
	Global activity	20.3 ± 1.1	23.4 ± 1.0^a^	22.1 ± 1.1^a^	16.51	0.000
Eccentric	Rectus femoris	2.0 ± 0.3	1.9 ± 0.3	2.0 ± 0.3	0.25	0.780
	Biceps femoris	14.5 ± 1.3	16.5 ± 1.7	14.7 ± 1.4	3.11	0.055
	Semitendinosus	16.5 ± 1.3	18.1 ± 1.2^a^	18.3 ± 1.3^a^	4.73	0.014
	Gluteus maximus	8.6 ± 1.0	8.3 ± 0.8	8.6 ± 1.0	0.19	0.822
	Gastrocnemius medialis	24.4 ± 1.8	29.9 ± 1.9^a^	27.5 ± 1.9	8.91	0.001
	Gastrocnemius lateralis	37.6 ± 3.2	39.0 ± 2.9	36.4 ± 2.8	1.24	0.198
	Global activity	17.3 ± 0.9	18.9 ± 0.9^a^	17.9 ± 0.9	7.39	0.002

*Data presented as normalized muscle activity (%MVIC); SE, standard error of the mean; Global activity, mean of the six muscles; ^*a*^significantly different with non-vibration condition; ^*b*^significantly different with vibration at 40 Hz condition.*

**TABLE 2:** The sEMG activity for each analyzed muscle under suspended hamstring curl conditions.

		Suspended hamstring curl				
Exercise phase	Muscle group	Non-Vibration	Vibration at 25 Hz	Vibration at 40 Hz	F	*p*
		Mean ± SE	Mean ± SE	Mean ± SE		
Concentric	Rectus femoris	1.3 ± 0.1	1.4 ± 0.1	1.2 ± 0.1	1.13	0.330
	Biceps femoris	23.6 ± 1.4	23.7 ± 1.3	24.0 ± 1.6	0.04	0.955
	Semitendinosus	24.9 ± 1.7	26.2 ± 1.6	25.8 ± 1.7	0.72	0.490
	Gluteus maximus	12.7 ± 1.1	13.1 ± 1.4	12.9 ± 1.1	0.16	0.848
	Gastrocnemius medialis	37.0 ± 3.0	37.6 ± 2.0	40.8 ± 3.4	1.61	0.210
	Gastrocnemius lateralis	52.8 ± 3.7	57.5 ± 3.8	56.2 ± 3.9	1.88	0.165
	Global activity	25.4 ± 1.1	26.5 ± 1.0	26.8 ± 1.2	2.60	0.086
Eccentric	Rectus femoris	1.4 ± 0.2	1.5 ± 0.2	1.8 ± 0.3	1.14	0.329
	Biceps femoris	22.0 ± 1.4	24.5 ± 1.7	22.6 ± 1.6	1.61	0.211
	Semitendinosus	20.6 ± 1.1	22.9 ± 1.5	22.5 ± 1.9	2.01	0.146
	Gluteus maximus	10.0 ± 0.8	11.7 ± 1.1	11.4 ± 1.0	3.48	0.060
	Gastrocnemius medialis	36.3 ± 2.1	37.0 ± 2.2	37.1 ± 2.2	0.17	0.838
	Gastrocnemius lateralis	51.5 ± 3.7	50.8 ± 3.6	51.2 ± 4.4	0.06	0.940
	Global activity	23.6 ± 0.9	24.7 ± 1.0	24.4 ± 1.1	1.85	0.169

*Data presented as normalized muscle activity (%MVIC); SE, standard error of the mean; Global activity, mean of the six muscles.*

### Suspended Supine Bridge

**FIGURE 3:**
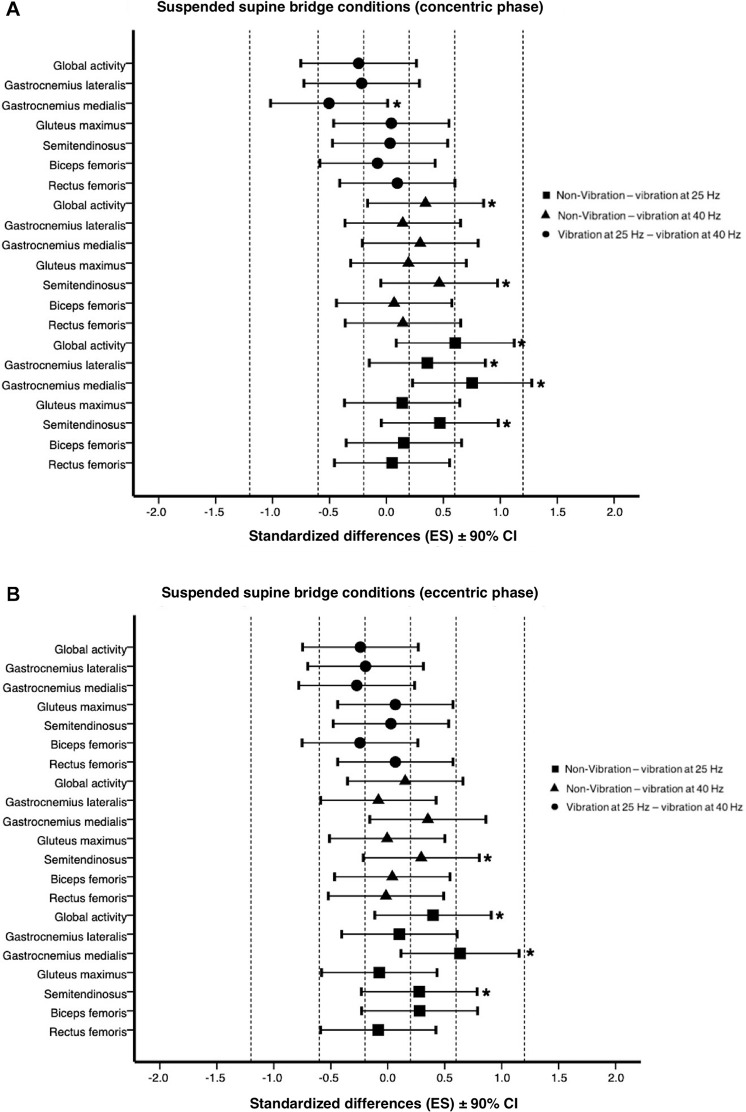
Effects of suspended supine bridge conditions on muscle activity (%MVIC) at concentric **(A)** and eccentric phase **(B)** expressed as standardized differences (Cohen’s *d*) ± 90% CI. Dotted line represents the effect size thresholds. * Significant differences at *p* < 0.05. ES, effect size; CI, confidence interval.

Supplementary Tables 3–5 shows the linear mixed model results. A significant fixed effect for exercise condition indicated that during the concentric phase, the suspended supine bridge with 25 Hz vibration showed a small increase with non-vibration condition for semitendinosus (*p* = 0.003, *d* = 0.47), gastrocnemius lateralis (*p* = 0.008, *d* = 0.36), and global activity (*p* = 0.000, *d* = 0.60). Moreover, the aforementioned conditions presented a moderate increase for gastrocnemius medialis (non-vibration vs 25 Hz vibration: *p* = 0.000, *d* = 0.75). The suspended supine bridge with 25 Hz vibration showed a small decrease with vibration at 40 Hz condition for gastrocnemius medialis (*p* = 0.025, *d* = −0.50). The semitendinosus and global activity showed a small increase between suspended supine bridge with 40 Hz vibration and non-vibration (*p* = 0.001, *d* = 0.46; *p* = 0.005, *d* = 0.34, respectively). For eccentric phase, the suspended supine bridge with 25 Hz vibration showed a small increase with non-vibration condition for semitendinosus (*p* = 0.046, *d* = 0.28) and global activity (*p* = 0.001, *d* = 0.40) and a moderate increase for gastrocnemius medialis (*p* = 0.000, *d* = 0.63). Additionally, the suspended supine bridge with 40 Hz vibration presented a small increase with non-vibration condition for semitendinosus (*p* = 0.024, *d* = 0.29). The standardized differences, expressed as Cohen *d* effect size, between exercise condition and muscle activity are shown detailed in Figure 3.

### Suspended Hamstring Curl

**FIGURE 4:**
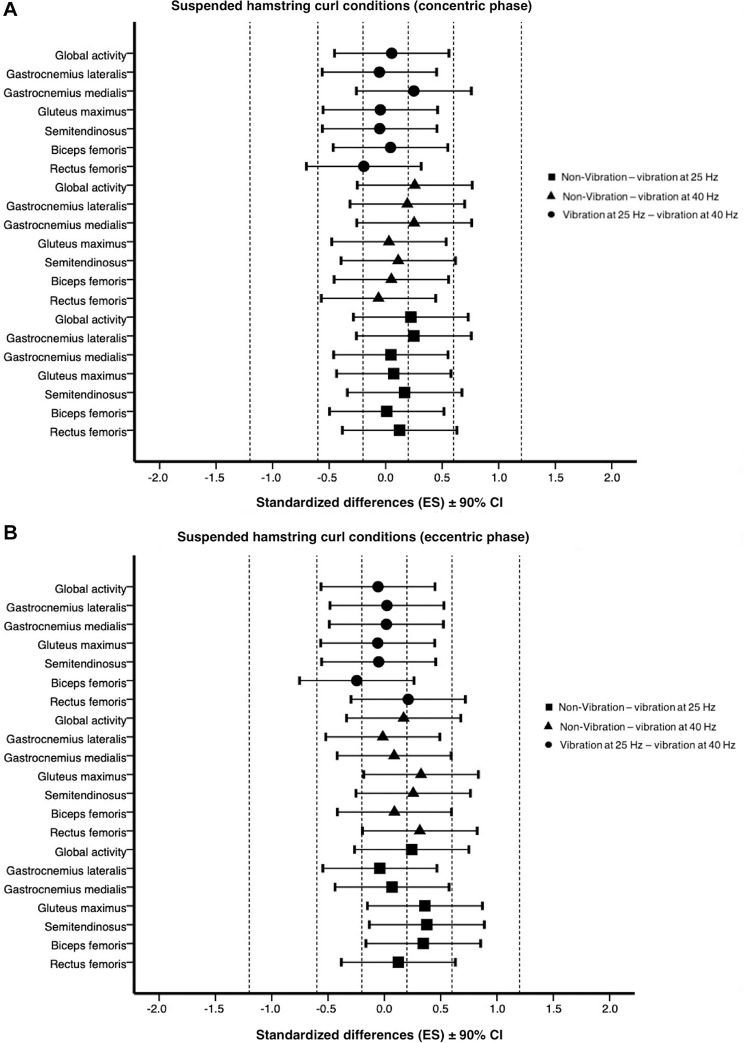
Effects of suspended hamstring curl conditions on muscle activity (%MVIC) at concentric **(A)** and eccentric phase **(B)** expressed as standardized differences (Cohen’s *d*) ± 90% CI. Dotted line represents the effect size thresholds. ES, effect size; CI, confidence interval.

The linear mixed model results are shown in Supplementary Tables 6–8. A non-significant fixed effect for exercise condition during the concentric phase neither eccentric phase was found on the analyzed muscles (Table 2). Additionally, the effect size analysis is shown in Figure 4.

### OMNI-Perceived Exertion Scale for Resistance Exercise

**FIGURE 5:**
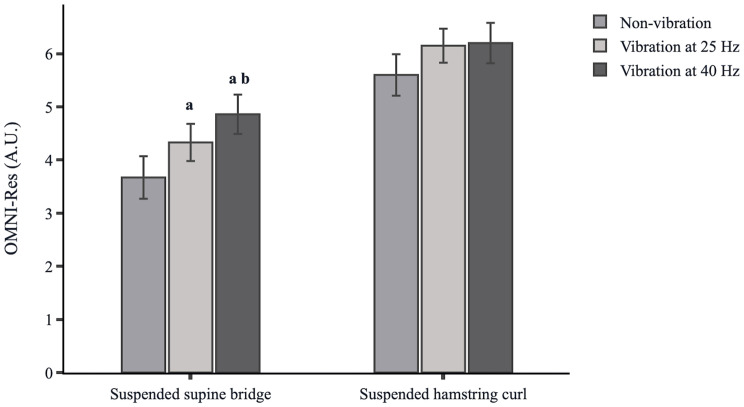
OMNI-Res (mean ± SE) for suspended supine bridge and suspended hamstring curl under non-vibration, vibration at 25 Hz and vibration at 40 Hz conditions. Each bar represents the mean, and the error bar represent the standard error of the mean (SE). A.U., Arbitrary units; ^a^significantly different with non-vibration condition; ^b^significantly different with vibration at 25 Hz condition.

Friedman test showed a significant main effect for suspended supine bridge [*X*^2^ (2) = 26.462, *p* = 0.000] but not for suspended hamstring curl [*X*^2^ (2) = 6.333, *p* = 0.052] on the OMNI-Res. Pairwise comparison showed a significantly higher OMNI-Res for suspended supine bridge with vibration at 40 Hz (4.86 ± 0.37) than for vibration at 25 Hz (4.33 ± 0.35, *p* = 0.024, *d* = 0.32 CI = −0.19, 0.83) and non-vibration condition (3.67 ± 0.40, *p* = 0.000, *d* = 0.67 CI = 0.15, 1.19). Moreover, OMNI-Res was significantly higher for suspended supine bridge with vibration at 25 Hz than for non-vibration condition (*p* = 0.003, *d* = 0.38 CI = −0.13, 0.89) (Figure 5). Supplementary Table 9 shows the percentage of change for the OMNI-Res under suspended supine bridge and suspended hamstring curl conditions.

## Discussion

Superimposed vibration in a suspension device increased lower limb muscle activity in the supine bridge but not in the hamstring curl exercise. In the suspended supine bridge, a significant moderate increase of 14.8% (concentric phase) and a small increase of 9.7% (eccentric phase) was found under the 25 Hz vibration condition compared to the non-vibration global activity. Likewise, 40 Hz vibration significantly increased global activation by 8.7% (a small increase) during the concentric phase. Similarly, Marín and Hazell (2014) applied superimposed 30 Hz vibration on an unstable surface (BOSU) and found a higher muscle activity between 23.5% and 35% in the isometric half-squat compared to the unstable condition. The effect of additional vibration (30 Hz and 40 Hz with an amplitude of 4 mm) on unstable surfaces and suspension devices increased the demands of the exercise. Thus, eliciting a greater activation of the lower limb muscles (vastus medialis and lateralis, biceps femoris, and gluteus medius) during the suspended lunge combined with 40 Hz WBV than in unstable or suspended exercises without vibration (Aguilera-Castells et al., 2019). Understanding what exercises generate more muscle activation and under what conditions they do so is essential for practitioners. Previous scientific research reveals that different tasks involving the same muscle groups can present significantly different activation levels (Malliaropoulos et al., 2015); these findings are relevant in injury prevention and rehabilitation.

The effect of two different frequencies was studied in the present study, finding a small to moderate significant increase in semitendinosus, gastrocnemius medialis, and lateralis activation under 25 Hz vibration compared to the non-vibration condition. Likewise, there was a significantly small decrease in the gastrocnemius medialis activity at 40 Hz (Figure 3). Furthermore, no significant differences were found among frequencies for the other analyzed muscles. Overall, this study showed that performing the 25 Hz suspended supine bridge elicits a greater activation than at 40 Hz vibration in almost all the analyzed muscles. In the same vein, a progressive increase in vibration frequency (5 Hz to 30 Hz) gradually enhanced the neuromuscular response for the lower limb muscles (soleus, gastrocnemius, tibialis anterior, biceps femoris, vastus medialis, and rectus femoris), achieving the highest activations at 25 to 30 Hz frequencies (Ritzmann et al., 2013). On the other hand, 25 Hz vibration was consistently more demanding than 40 Hz vibration [concentric phase: biceps femoris (−3.0%, trivial), gastrocnemius medialis (−12.2%, small decrease), gastrocnemius lateralis (−7.4%, small decrease), global activity (−5.2%, small decrease); eccentric phase: biceps femoris (−10. 5%, small decrease), gastrocnemius medialis: (−8.0%, small decrease), gastrocnemius lateralis (−6.6%, trivial), global activity (−5.4%, small decrease)], per Cardinale and Lim (2003), who found lower but not significant muscle activity of 40 Hz vibration compared to 30 Hz. Regarding the effect of the different frequencies on the analyzed muscles, higher activation was found for the more proximal muscles exposed to the vibration. The additional effect of vibration at 25 Hz compared to the non-vibration suspended condition was significantly higher for the gastrocnemius (medialis and lateralis) and semitendinosus in the concentric and eccentric phase (from 9.8% to 23.8% with trivial to moderate effect). Previous studies also demonstrated that the more proximal to the vibration experimented higher activities than the more distal muscles (Hazell et al., 2010; Ritzmann et al., 2013). In this regard, the present study showed that in both vibration conditions (25 Hz and 40 Hz), the muscle excitation sequence (Neto et al., 2019), from higher to lower activation, was gastrocnemius lateralis, gastrocnemius medialis, semitendinosus, biceps femoris, gluteus maximus, and rectus femoris (Table 1). Thus, the magnitude of the neuromuscular response to the vibratory stimulus in those muscles that are closer to the most proximal joints (ankles) dissipates the effects of vibration for the more distal muscles, acting as a damper (Abercromby et al., 2007b). Indeed, the vibration induces different reflexes that favor increased muscle activation on the most proximal muscles, such as the tonic vibration reflex (Issurin, 2005; Ritzmann et al., 2010) or the stretch reflex on the soft tissues (Cardinale and Lim, 2003; Cochrane et al., 2009).

Of all analyzed muscles, gastrocnemius lateralis (41–60% MVIC) achieved a high activation under 25 Hz vibration and slightly lower (37.4% MVIC) for gastrocnemius medialis. Participants were asked to perform an ankle plantar flexion on the strap cradles instead of leaning their heels on the suspension cradles in the suspended supine bridge. Ritzmann et al. (2013) found that the variation of the foot position on the vibration platform increased the gastrocnemius medialis activity up to 48% (forefoot stance vs. normal stance). Although the feet remained in plantar flexion in the three suspended supine bridge conditions in the current study, the percentage of gastrocnemius activity significantly increased (14–23%, from small to moderate increase) under 25 Hz vibration to the non-vibration condition. The lack of differences between the 40 Hz vibration and the non-vibration suspended condition could be explained because gastrocnemius is more predominantly activated at frequencies below 40 Hz (20, 25, and 30 Hz) (Di Giminiani et al., 2013), according to the findings of the present study (Table 1).

The hamstrings (biceps femoris and semitendinosus) muscle activity ranged from moderate to low (< 24% MVIC), with significant differences in semitendinosus activity at 25 Hz and 40 Hz in comparison to the non-vibration condition. However, following Abercromby et al. (2007a), the biceps femoris activity was slightly lower, with similar activation in all conditions. This low activation (< 21% MVIC) of the biceps femoris is related to 90° knee flexion in the suspended supine bridge. Ho et al. (2020) found a similar low activation (18% MVIC) of the biceps femoris in the dynamic supine bridge (90° knee flexion). However, the effect of WBV in the static supine bridge, maintaining the 90° of knee flexion, elicited a significant moderate activation (21–40% MVIC) of the biceps femoris at 30 Hz and 50 Hz, although the non-vibration condition also showed a moderate level of activation (27% MVIC). The authors supported that 50 Hz vibration was more demanding for the biceps femoris in the static supine bridge (Marín and Cochrane, 2021). Similarly, Hazell et al. (2007) found an increase in biceps femoris activation between 35 Hz and 45 Hz for dynamic and static squats. This suggested that superimposed vibration (25 Hz and 40 Hz) in the dynamic suspended supine bridge is insufficient to significantly stimulate the biceps femoris compared to the non-vibration condition significantly. Thus, an increased frequency of superimposed vibration on the suspension straps (> 40 Hz) and performing the exercise unilaterally, single-leg suspended supine bridge, could increase the demand of the biceps femoris to high activations (> 41% MVIC), as indicated by previous studies on sEMG on the single-leg supine bridge on the floor (Lehecka et al., 2017), or on a BOSU (Youdas et al., 2015). In this vein, the functional magnetic resonance imaging study conducted by Bourne et al. (2018b) found a predominant activation of the biceps femoris long head. Likewise, there could be several reasons for the small differences between the biceps femoris and semitendinosus in the suspended supine bridge. One reason is that the suspended exercise produces lateral instability, provoking a lateral rotation of the thighs and, consequently, an increased semitendinosus activity because of its role in counteracting this movement (Tobey and Mike, 2018). Furthermore, the amplitude of the vibrating machine (8mm, peak to peak) is suggested to provoke more horizontal oscillations and focus on the stabilizing structures that, in the present study, are stabilized by the semitendinosus (Cook et al., 2011). Another reason is that the necessity to keep the feet stable and maintain the anchor in a plumb line (perpendicular to the ground) of the suspension strap requires the participation of the posterior thigh muscles, similar to the feet-away hip thrust (Collazo García et al., 2020). This semi-stretched position provokes an increase in muscle tension and enhances the effects of the vibration in the hamstrings muscles (Cardinale and Lim, 2003; Marín and Cochrane, 2021). Overall, as a practical application, muscles with activations below 45% MVIC, such as biceps femoris and semitendinosus in suspended supine bridge conditions (non-vibration, 25 Hz and 40 Hz vibrations), would be targeted for muscular endurance, stabilization, and rehabilitation training programs (Ekstrom et al., 2007; Youdas et al., 2015).

Although the barbell hip thrust is a very demanding exercise for gluteus maximus (> 60% MVIC) (Neto et al., 2019), the variation of suspended (and unloaded) exercise proposed in this study elicited low activation (< 23% MVIC) with a trivial and small effect among conditions (Figure 3). In this vein, previous studies have reported activation levels ranging from moderate to low (< 25% MVIC) for gluteus maximus in unloaded supine bridge on the floor (Ekstrom et al., 2007; Jang et al., 2013; Kim and Park, 2016). Thus, it appears that the suspended supine bridge (with an additional effect of vibration) is as demanding for the gluteus maximus as the traditional supine bridge exercise and are not sufficiently challenged to reach high and very high activation values (> 40% MVIC) in the gluteus maximus, as happens with the single-leg bridge (Ekstrom et al., 2007; Lehecka et al., 2017), the WBV supine bridge (Marín and Cochrane, 2021) or the barbell hip thrust (Contreras et al., 2016; Andersen et al., 2018; Williams et al., 2021). Therefore, although the gluteus maximus is the prime supine bridge mover, its activation is still low. Moreover, superimposed vibrations were dampened by the more proximal to vibration musculature, and the gluteus maximus were not overstimulated. In addition, the rectus femoris showed the lowest activation (< 2.0% MVIC) with a trivial effect in both phases of exercise without significant differences among conditions. Collazo García et al. (2020) showed a significantly (2.4%) lower rectus femoris activation in the feet-away barbell hip thrust (3.4% MVIC) compared to the original hip thrust condition (5.8% MVIC). Likewise, Lehecka et al. (2017) found similar rectus femoris activity in the unloaded single-leg bridge with 90° of knee flexion, agreeing with the present study results.

Conversely, as hypothesized, the additional effect of the superimposed vibration did not result in a significantly higher activation in any of the analyzed muscles, or the global activity, during the concentric and eccentric phases of the suspended hamstring curl (Table 2). Moreover, differences among exercise conditions ranged from trivial to small (Figure 4). Even though the muscle excitation sequence was similar to the suspended supine bridge. Thus, the activation increments of the most proximal muscles to the vibratory stimulus (gastrocnemius medialis and lateralis) were between 9% and 5% ranged from trivial to small increase in 25 Hz and 40 Hz vibration, respectively, to the non-vibration condition. The main difference in transmitting the vibration between the suspended supine bridge and the suspended hamstring curl was the suspension strap position. The straps remained in a plumb line in the supine bridge, whereas it acted as a pendulum in the suspended hamstring curl. Several studies suggested that vibration transmission via cable in pulley exercises such as biceps curl or one arm pulleying keep the perpendicular between the anchor point, vibration device, and handle to enhance the effects of local vibration (Bosco et al., 1999; Issurin and Tenenbaum, 1999; Issurin et al., 2012). Nevertheless, the pendulum motion in the suspended hamstring curl could attenuate vibration transmission because the vibratory system is designed to transmit the vibration. Moreover, it could be speculated that the pendulum motion could also exert a dampening effect by inhibiting the tonic vibratory reflex (Rittweger, 2010). On the other hand, the pendulum motion and plantar flexion to keep the feet on the cradles could explain the gastrocnemius activity in the suspended hamstring curl conditions. Additionally, Bettendorf (2010) suggested that the intensity variation in a suspended exercise is based on three fundamental principles. Thus, the pendulum principle could justify that the prime mover activations (biceps femoris and semitendinosus) in this study were slightly higher than low activations (< 21% MVIC) reported by Árnason et al. (2014) in the suspended hamstring curl without pendulum movement and lower than high and very high activations (> 50% MVIC) registered by Malliaropoulos et al. (2015) in the suspended hamstring curl with alternating knee flexion and pendulum motion.

Regarding OMNI-Res, the finding was that superimposed vibration increased the value of subjective perception of exertion compared to the non-vibration suspended condition around 10% (small increase) for both vibration frequencies in the suspended hamstring curl and from 18% to 32% (small to moderate increase) for the suspended supine bridge. Thus, it seems that the value of OMNI-Res increases progressively while increasing the vibration frequency, being consistent with the significant correlation (*r* = 0.95) between OMNI-Res and a range of vibration frequency (25 Hz to 45 Hz) and amplitudes (1 and 3 mm) found by Marín et al. (2011). Additionally, the validity and reliability of the intensity of exertion using subjective scales in exercises with superimposed vibration have been demonstrated for both vibration frequency and muscle activation (Marín et al., 2012).

There were some limitations in the study. The effect of superimposed vibration on suspended exercises has been assessed in physically active men and women, so the results obtained in the present study cannot be generalized to other populations. The footwear soles were different among participants, and since this area is the most exposed to vibration, this could slightly modify the vibratory stimulus due to the damping effect of the footwear soles. Therefore, future research should standardize the footwear for all participants. Likewise, the vibration transmitted through the suspension strap could have dissipated the vibration effect. While the distance between the suspension strap and the ground was standardized, it could be interesting to examine different suspension strap heights and their effects on muscle demand in the supine bridge in future studies. Another limitation was that the erector spinae and vastus (medialis and lateralis) requested in the supine bridge were not evaluated because the electromyography system employed only offers six channels. Further investigations could study the effects of superimposed vibration on neuromuscular performance in a loaded suspended supine bridge (kettlebell, barbells, weight plates) or variations of the exercise such as a single-leg or modifying the arm positions (crossed over the chest).

## Conclusion

The additional effect of the superimposed vibration resulted in being more challenging for the suspended supine bridge than the suspended hamstring curl. Although the two vibration frequencies elicit the same activation level at the global activity level, the suspended supine bridge with a 25 Hz vibration provoked a higher activity of the most proximal muscles to the vibration device (gastrocnemius medialis, lateralis, and semitendinosus), with meaningless effects on the primary movers. Therefore, the amount of instability provoked by the suspended supine bridge with superimposed vibration increased the stabilizing role of the gastrocnemius and semitendinosus. In contrast, the anteroposterior movement of the suspended hamstring exercise seems to be less effective in transmitting the vibration. Regardless of the exercise, increasing the vibration frequency on the suspension device leads to a higher value of subjective perception of exertion (OMNI-Res).

### Practical Application

The suspended supine bridge is as demanding as a traditional exercise for the gluteus maximus. However, the additional effect of the superimposed vibration in the suspended supine bridge provides greater gastrocnemius and hamstrings activity. Plantar flexion in the suspended supine bridge with superimposed vibration is a successful manner for strengthening the gastrocnemius, demanded in sports actions such as changes of direction, jumps, and sprints. Furthermore, this method allows dynamic tasks, changing the planes of the force production and offering a continuous exposition to vibration for the working muscles. Likewise, the increased instability generated through vibration to the suspension straps turns the suspended supine bridge into an exercise that demands the neutralization of the lateral rotation of the thighs, similar to other lateral actions in several sports actions. Moreover, superimposed vibration in a suspension device can complement traditional exercises such as the Nordic hamstring, leg curl, or deadlift to develop the strength and endurance of the hamstrings in strength and conditioning programs. Additionally, injury prevention and rehabilitation can benefit from the outputs of the present study to further evaluate the inclusion of superimposed vibration in the prescribed protocols since hamstrings injuries are prevalent in many sports.

## Data Availability Statement

The datasets presented in this study can be found in online repositories. The names of the repository/repositories and accession number(s) can be found below: Figshare (https://doi.org/10.6084/m9.figshare.14537001).

## Ethics Statement

The studies involving human participants were reviewed and approved by Ethics and Research Committee Board in the Blanquerna Faculty of Psychology and Educational and Sport Sciences at Ramon Llull University in Barcelona, Spain, with reference number 1819005D. The patients/participants provided their written informed consent to participate in this study. Written informed consent was obtained from the individual(s) for the publication of any potentially identifiable images or data included in this article.

## Author Contributions

JA-C, BB, AF-V, and JP contributed to the conception and design of the study. JA-C, JA-A, AF-V, and AM contributed to the acquisition of data. JA-C, JA-A, and AM analyzed the data. JA-C and BB wrote the original draft of the manuscript. BB and JP supervised the study. All authors wrote, edited, reviewed, and approved the submitted final version of the manuscript.

## Conflict of Interest

JA-C and BB are affiliated to the Faculty of Psychology, Education Sciences, and Sport Blanquerna, of the Ramon Llull University, and JP is affiliated to the University of Vic - Central University of Catalonia, which have requested for a patent (number 202030652, Spanish Patent and Trademark Office - OEPM) enabling the use of superimposed vibration in athletic, fitness, and health settings. The remaining authors declare that the research was conducted in the absence of any commercial or financial relationships that could be construed as a potential conflict of interest.

## Publisher’s Note

All claims expressed in this article are solely those of the authors and do not necessarily represent those of their affiliated organizations, or those of the publisher, the editors and the reviewers. Any product that may be evaluated in this article, or claim that may be made by its manufacturer, is not guaranteed or endorsed by the publisher.
