# Supplementary material for: sEMG Activity in Superimposed Vibration on Suspended Supine Bridge and Hamstring Curl
Source: Front Physiol. 2021 Aug 11;12:712471. doi: 10.3389/fphys.2021.712471 (PMC8385437; doi:10.3389/fphys.2021.712471)
Supplement: Supplementary file 1 [file Table_1.DOCX]

|  | | **Suspended supine bridge** | | | | |
| --- | --- | --- | --- | --- | --- | --- |
| **Exercise phase** | **Muscle group** | **Non-vibro vs vibro 25** |  | **Non-vibro vs vibro 40** |  | **Vibro 25 vs vibro 40** |
|  |  | **%** |  | **%** |  | **%** |
| Concentric | Rectus femoris | 4,79 |  | 16,49 |  | 11,16 |
|  | Biceps femoris | 6,03 |  | 2,79 |  | -3,05 |
|  | Semitendinosus | 16,35 |  | 17,58 |  | 1,06 |
|  | Gluteus maximus | 8,93 |  | 11,99 |  | 2,81 |
|  | Gastrocnemius medialis | 23,84 |  | 8,66 |  | -12,26 |
|  | Gastrocnemius lateralis | 14,16 |  | 5,62 |  | -7,48 |
|  | Global activity | 14,87 |  | 8,79 |  | -5,29 |
| Eccentric | Rectus femoris | -6,97 |  | -1,32 |  | 6,07 |
|  | Biceps femoris | 13,77 |  | 1,76 |  | -10,56 |
|  | Semitendinosus | 9,87 |  | 10,86 |  | 0,90 |
|  | Gluteus maximus | -3,66 |  | -0,28 |  | 3,51 |
|  | Gastrocnemius medialis | 22,33 |  | 12,54 |  | -8,01 |
|  | Gastrocnemius lateralis | 3,88 |  | -3,06 |  | -6,68 |
|  | Global activity | 9,72 |  | 3,77 |  | -5,43 |

**Supplementary Table 1.** Percentage of change for each analyzed muscle under suspended supine bridge conditions.
