# Supplementary material for: sEMG Activity in Superimposed Vibration on Suspended Supine Bridge and Hamstring Curl
Source: Front Physiol. 2021 Aug 11;12:712471. doi: 10.3389/fphys.2021.712471 (PMC8385437; doi:10.3389/fphys.2021.712471)
Supplement: Supplementary file 2 [file Table_2.DOCX]

|  | | **Suspended hamstring curl** | | | | |
| --- | --- | --- | --- | --- | --- | --- |
| **Exercise phase** | **Muscle group** | **Non-vibro vs vibro 25** |  | **Non-vibro vs vibro 40** |  | **Vibro 25 vs vibro 40** |
|  |  | **%** |  | **%** |  | **%** |
| Concentric | Rectus femoris | 6,55 |  | -2,94 |  | -8,91 |
|  | Biceps femoris | 0,25 |  | 1,51 |  | 1,25 |
|  | Semitendinosus | 5,17 |  | 3,53 |  | -1,56 |
|  | Gluteus maximus | 3,42 |  | 1,20 |  | -2,14 |
|  | Gastrocnemius medialis | 1,47 |  | 10,15 |  | 8,55 |
|  | Gastrocnemius lateralis | 8,26 |  | 6,38 |  | -1,74 |
|  | Global activity | 4,45 |  | 5,56 |  | 1,07 |
| Eccentric | Rectus femoris | 8,88 |  | 29,35 |  | 18,80 |
|  | Biceps femoris | 11,67 |  | 2,86 |  | -7,89 |
|  | Semitendinosus | 11,20 |  | 9,20 |  | -1,80 |
|  | Gluteus maximus | 17,06 |  | 14,06 |  | -2,56 |
|  | Gastrocnemius medialis | 1,91 |  | 2,41 |  | 0,49 |
|  | Gastrocnemius lateralis | -1,31 |  | -0,52 |  | 0,80 |
|  | Global activity | 4,74 |  | 3,50 |  | -1,19 |

**Supplementary Table 2.** Percentage of change for each analyzed muscle under suspended hamstring curl conditions
