# Supplementary material for: sEMG Activity in Superimposed Vibration on Suspended Supine Bridge and Hamstring Curl
Source: Front Physiol. 2021 Aug 11;12:712471. doi: 10.3389/fphys.2021.712471 (PMC8385437; doi:10.3389/fphys.2021.712471)
Supplement: Supplementary file 3 [file Table_3.DOCX]

| **Suspended supine bridge: concentric phase** | | | | | | | |
| --- | --- | --- | --- | --- | --- | --- | --- |
|  | **Parameter** | **ES** | **SE** | **95%CI**  **0.60-0.85**  **-0.17-0.01**  **-0.19-0.03**  **-0.05-0.11** | | **t** | **p** |
|  |  |  |  | Lower | Upper |  |  |
| **Rectus Femoris** | Intercept | 2.08 | 0.42 | 1.22 | 2.93 | 4.91 | 0.00 |
|  | Non-vibration | -0.29 | 0.47 | -1.25 | 0.66 | -0.62 | 0.54 |
|  | Vibration at 25 Hz | -0.21 | 0.47 | -1.16 | 0.74 | -0.44 | 0.66 |
|  | σ_u_ | 1.19 | | | | | |
|  | σ_є_ | 1.53 | | | | | |
|  |  |  |  |  | |  |  |
| **Biceps femoris** | Intercept | 19.63 | 1.65 | 16.24 | 23.03 | 11.88 | 0.00 |
|  | Non-vibration | -0.53 | 0.96 | -2.46 | 1.39 | -0.56 | 0.58 |
|  | Vibration at 25 Hz | 0.62 | 0.96 | -1.31 | 2.54 | 0.65 | 0.52 |
|  | σ_u_ | 6.91 | | | | | |
|  | σ_є_ | 3.09 | | | | | |
|  |  |  |  |  | |  |  |
| **Semitendinosus** | Intercept | 23.22 | 1.56 | 20.01 | 26.43 | 14.86 | 0.00 |
|  | Non-vibration | -3.47 | 0.91 | -5.31 | -1.63 | -3.81 | 0.00 |
|  | Vibration at 25 Hz | -0.24 | 0.91 | -2.08 | 1.59 | -0.27 | 0.79 |
|  | σ_u_ | 6.52 | | | | | |
|  | σ_є_ | 2.95 | | | | | |
|  |  |  |  |  | |  |  |
| **Gluteus maximus** | Intercept | 16.62 | 2.09 | 12.31 | 20.92 | 7.96 | 0.00 |
|  | Non-vibration | -1.77 | 0.98 | -3.74 | 0.18 | -1.82 | 0.75 |
|  | Vibration at 25 Hz | -0.45 | 0.98 | -2.42 | 1.51 | -0.47 | 0.64 |
|  | σ_u_ | 9.03 | | | | | |
|  | σ_є_ | 3.16 | | | | | |
|  |  |  | | | | | |
| **Gastrocnemius medialis** | Intercept | 32.85 | 1.95 | 28.88 | 36.82 | 16.82 | 0.00 |
|  | Non-vibration | -2.62 | 1.66 | -5.95 | 0.72 | -1.58 | 0.12 |
|  | Vibration at 25 Hz | 4.59 | 1.66 | 1.24 | 7.93 | 2.77 | 0.01 |
|  | σ_u_ | 7.17 | | | | | |
|  | σ_є_ | 5.36 | | | | | |
|  |  |  | | | | | |
| **Gastrocnemius lateralis** | Intercept | 38.59 | 3.06 | 32.30 | 44.89 | 12.62 | 0.00 |
|  | Non-vibration | -2.05 | 1.62 | -5.32 | 1.21 | -1.27 | 0.21 |
|  | Vibration at 25 Hz | 3.12 | 1.62 | -0.14 | 6.39 | 1.93 | 0.04 |
|  | σ_u_ | 7.42 | | | | | |
|  | σ_є_ | 2.44 | | | | | |

**Supplementary Table 3.** Linear mixed model for suspended supine bridge conditions (concentric phase) with muscle activity as the dependent variable.

ES = coefficient estimate; SE = standard error; 95% CI = 95% confidence intervals; t = t- value; p = p-value; σ_u_ = standard deviation of participant; σ_є_ = standard deviation of residual. The “suspended supine bridge with vibration at 40 Hz” was used as reference categories for this model in the exercise condition variable.
