# Supplementary material for: sEMG Activity in Superimposed Vibration on Suspended Supine Bridge and Hamstring Curl
Source: Front Physiol. 2021 Aug 11;12:712471. doi: 10.3389/fphys.2021.712471 (PMC8385437; doi:10.3389/fphys.2021.712471)
Supplement: Supplementary file 4 [file Table_4.DOCX]

| **Suspended supine bridge: eccentric phase** | | | | | | | |
| --- | --- | --- | --- | --- | --- | --- | --- |
|  | **Parameter** | **ES** | **SE** | **95%CI**  **0.60-0.85**  **-0.17-0.01**  **-0.19-0.03**  **-0.05-0.11** | | **t** | **p** |
|  |  |  |  | Lower | Upper |  |  |
| **Rectus Femoris** | Intercept | 2.02 | 0.37 | 1.26 | 2.77 | 5.51 | 0.00 |
|  | Non-vibration | 0.03 | 0.21 | -0.40 | 0.46 | 0.13 | 0.90 |
|  | Vibration at 25 Hz | -0.12 | 0.21 | -0.55 | 0.32 | -0.54 | 0.59 |
|  | σ_u_ | 1.53 | | | | | |
|  | σ_є_ | 0.69 | | | | | |
|  |  |  |  |  | |  |  |
| **Biceps femoris** | Intercept | 14.78 | 1.47 | 11.76 | 17.80 | 10.04 | 0.00 |
|  | Non-vibration | -0.26 | 0.87 | -2.01 | 1.51 | -0.29 | 0.77 |
|  | Vibration at 25 Hz | 1.75 | 0.87 | -0.02 | 3.51 | 2.00 | 0.05 |
|  | σ_u_ | 6.12 | | | | | |
|  | σ_є_ | 2.83 | | | | | |
|  |  |  |  |  | |  |  |
| **Semitendinosus** | Intercept | 18.31 | 1.28 | 15.70 | 20.92 | 14.44 | 0.00 |
|  | Non-vibration | -1.79 | 0.64 | -3.09 | -0.49 | -2.78 | 0.01 |
|  | Vibration at 25 Hz | -0.16 | 0.64 | -1.46 | 1.13 | -0.25 | 0.80 |
|  | σ_u_ | 5.42 | | | | | |
|  | σ_є_ | 2.08 | | | | | |
|  |  |  |  |  | |  |  |
| **Gluteus maximus** | Intercept | 8.62 | 0.95 | 6.65 | 10.58 | 9.02 | 0.00 |
|  | Non-vibration | 0.02 | 0.56 | -1.10 | 1.15 | 0.04 | 0.97 |
|  | Vibration at 25 Hz | -0.29 | 0.56 | -1.42 | 0.84 | -0.52 | 0.61 |
|  | σ_u_ | 3.98 | | | | | |
|  | σ_є_ | 1.81 | | | | | |
|  |  |  | | | | | |
| **Gastrocnemius medialis** | Intercept | 27.50 | 1.85 | 23.71 | 31.29 | 14.84 | 0.00 |
|  | Non-vibration | -3.06 | 1.30 | -5.68 | -0.45 | -2.37 | 0.02 |
|  | Vibration at 25 Hz | 2.39 | 1.30 | -0.22 | 5.01 | 1.85 | 0.07 |
|  | σ_u_ | 7.38 | | | | | |
|  | σ_є_ | 4.19 | | | | | |
|  |  |  | | | | | |
| **Gastrocnemius lateralis** | Intercept | 36.47 | 2.96 | 30.40 | 42.55 | 12.34 | 0.00 |
|  | Non-vibration | 1.15 | 1.66 | -2.19 | 4.49 | 0.69 | 0.49 |
|  | Vibration at 25 Hz | 2.61 | 1.66 | -0.73 | 5.95 | 1.58 | 0.12 |
|  | σ_u_ | 12.43 | | | | | |
|  | σ_є_ | 5.37 | | | | | |

**Supplementary Table 4.** Linear mixed model for suspended supine bridge conditions (eccentric phase) with muscle activity as the dependent variable.

ES = coefficient estimate; SE = standard error; 95% CI = 95% confidence intervals; t = t- value; p = p-value; σ_u_ = standard deviation of participant; σ_є_ = standard deviation of residual. The “suspended supine bridge with vibration at 40 Hz” was used as reference categories for this model in the exercise condition variable.
