# Supplementary material for: sEMG Activity in Superimposed Vibration on Suspended Supine Bridge and Hamstring Curl
Source: Front Physiol. 2021 Aug 11;12:712471. doi: 10.3389/fphys.2021.712471 (PMC8385437; doi:10.3389/fphys.2021.712471)
Supplement: Supplementary file 5 [file Table_5.DOCX]

| **Suspended supine bridge: concentric phase** | | | | | | | |
| --- | --- | --- | --- | --- | --- | --- | --- |
|  | **Parameter** | **ES** | **SE** | **95%CI**  **0.60-0.85**  **-0.17-0.01**  **-0.19-0.03**  **-0.05-0.11** | | **t** | **p** |
|  |  |  |  | Lower | Upper |  |  |
| **Global activity** | Intercept | 22.17 | 1.09 | 19.93 | 24.40 | 20.40 | 0.00 |
|  | Non-vibration | -1.79 | 0.53 | -2.86 | -0.72 | -3.38 | 0.00 |
|  | Vibration at 25 Hz | 1.23 | 0.53 | 0.17 | 2.31 | 2.34 | 0.02 |
|  | σ_u_ | 4.67 | | | | | |
|  | σ_є_ | 1.71 | | | | | |
| **Suspended supine bridge: eccentric phase** | | | | | | | |
|  | **Parameter** | **ES** | **SE** | **95%CI** | | **t** | **p** |
|  |  |  |  | Lower | Upper |  |  |
| **Global activity** | Intercept | 17.95 | 0.91 | 16.08 | 19.82 | 19.80 | 0.00 |
|  | Non-vibration | -0.65 | 0.44 | -1.54 | 0.24 | -1.48 | 0.15 |
|  | Vibration at 25 Hz | 1.03 | 0.44 | 0.14 | 1.92 | 2.34 | 0.02 |
|  | σ_u_ | 3.90 | | | | | |
|  | σ_є_ | 1.43 | | | | | |

**Supplementary Table 5.** Linear mixed model for suspended supine bridge (concentric and eccentric phase) with global activity as the dependent variable.

ES = coefficient estimate; SE = standard error; 95% CI = 95% confidence intervals; t = t- value; p = p-value; σ_u_ = standard deviation of participant; σ_є_ = standard deviation of residual. The “suspended supine bridge with vibration at 40 Hz” was used as reference categories for this model in the exercise condition variable.
