# Supplementary material for: sEMG Activity in Superimposed Vibration on Suspended Supine Bridge and Hamstring Curl
Source: Front Physiol. 2021 Aug 11;12:712471. doi: 10.3389/fphys.2021.712471 (PMC8385437; doi:10.3389/fphys.2021.712471)
Supplement: Supplementary file 6 [file Table_6.DOCX]

| **Suspended hamstring curl: concentric phase** | | | | | | | |
| --- | --- | --- | --- | --- | --- | --- | --- |
|  | **Parameter** | **ES** | **SE** | **95%CI**  **0.60-0.85**  **-0.17-0.01**  **-0.19-0.03**  **-0.05-0.11** | | **t** | **p** |
|  |  |  |  | Lower | Upper |  |  |
| **Rectus Femoris** | Intercept | 1.29 | 0.14 | 1.00 | 1.57 | 9.18 | 0.00 |
|  | Non-vibration | 0.04 | 0.09 | -0.13 | 0.21 | 0.46 | 0.65 |
|  | Vibration at 25 Hz | 0.13 | 0.09 | -0.05 | 0.29 | 1.47 | 0.15 |
|  | σ_u_ | 0.76 | | | | | |
|  | σ_є_ | 0.27 | | | | | |
|  |  |  |  |  | |  |  |
| **Biceps femoris** | Intercept | 24.04 | 1.44 | 21.10 | 26.97 | 16.61 | 0.00 |
|  | Non-vibration | -0.36 | 1.26 | -2.90 | 2.19 | -0.28 | 0.78 |
|  | Vibration at 25 Hz | -0.29 | 1.26 | -2.84 | 2.25 | -0.24 | 0.82 |
|  | σ_u_ | 5.21 | | | | | |
|  | σ_є_ | 4.09 | | | | | |
|  |  |  |  |  | |  |  |
| **Semitendinosus** | Intercept | 25.82 | 1.65 | 22.43 | 29.21 | 15.58 | 0.00 |
|  | Non-vibration | -0.88 | 1.09 | -3.09 | 1.32 | -0.80 | 0.43 |
|  | Vibration at 25 Hz | 0.41 | 1.09 | -1.80 | 2.61 | 0.37 | 0.71 |
|  | σ_u_ | 6.71 | | | | | |
|  | σ_є_ | 3.55 | | | | | |
|  |  |  |  |  | |  |  |
| **Gluteus maximus** | Intercept | 12.90 | 1.23 | 10.37 | 15.43 | 10.45 | 0.00 |
|  | Non-vibration | -0.15 | 0.77 | -1.70 | 1.40 | -0.20 | 0.84 |
|  | Vibration at 25 Hz | 0.28 | 0.77 | -1.27 | 1.83 | 0.36 | 0.72 |
|  | σ_u_ | 5.08 | | | | | |
|  | σ_є_ | 2.49 | | | | | |
|  |  |  | | | | | |
| **Gastrocnemius medialis** | Intercept | 40.84 | 2.83 | 35.07 | 46.61 | 14.40 | 0.00 |
|  | Non-vibration | -3.76 | 2.26 | -8.32 | 0.80 | -1.67 | 0.10 |
|  | Vibration at 25 Hz | -3.22 | 2.26 | -7.78 | 1.34 | -1.42 | 0.16 |
|  | σ_u_ | 10.74 | | | | | |
|  | σ_є_ | 7.33 | | | | | |
|  |  |  | | | | | |
| **Gastrocnemius lateralis** | Intercept | 56.26 | 3.76 | 48.54 | 63.97 | 14.95 | 0.00 |
|  | Non-vibration | -3.37 | 2.36 | -8.13 | 1.39 | -1.43 | 0.16 |
|  | Vibration at 25 Hz | 0.99 | 2.36 | -3.77 | 5.76 | 0.42 | 0.68 |
|  | σ_u_ | 15.46 | | | | | |
|  | σ_є_ | 7.65 | | | | | |

**Supplementary Table 6.** Linear mixed model for suspended hamstring curl conditions (concentric phase) with muscle activity as the dependent variable.

ES = coefficient estimate; SE = standard error; 95% CI = 95% confidence intervals; t = t- value; p = p-value; σ_u_ = standard deviation of participant; σ_є_ = standard deviation of residual. The “suspended hamstring curl with vibration at 40 Hz” was used as reference categories for this model in the exercise condition variable.
