# Supplementary material for: sEMG Activity in Superimposed Vibration on Suspended Supine Bridge and Hamstring Curl
Source: Front Physiol. 2021 Aug 11;12:712471. doi: 10.3389/fphys.2021.712471 (PMC8385437; doi:10.3389/fphys.2021.712471)
Supplement: Supplementary file 7 [file Table_7.DOCX]

| **Suspended hamstring curl: eccentric phase** | | | | | | | |
| --- | --- | --- | --- | --- | --- | --- | --- |
|  | **Parameter** | **ES** | **SE** | **95%CI**  **0.60-0.85**  **-0.17-0.01**  **-0.19-0.03**  **-0.05-0.11** | | **t** | **p** |
|  |  |  |  | Lower | Upper |  |  |
| **Rectus Femoris** | Intercept | 1.84 | 0.27 | 1.30 | 2.37 | 6.90 | 0.00 |
|  | Non-vibration | -0.42 | 0.28 | -0.99 | 0.15 | -1.47 | 0.15 |
|  | Vibration at 25 Hz | -0.29 | 0.28 | -0.86 | 0.28 | -1.03 | 0.31 |
|  | σ_u_ | 0.81 | | | | | |
|  | σ_є_ | 0.92 | | | | | |
|  |  |  |  |  | |  |  |
| **Biceps femoris** | Intercept | 22.63 | 1.59 | 19.41 | 25.85 | 14.21 | 0.00 |
|  | Non-vibration | -0.63 | 1.49 | -3.63 | 2.37 | -0.42 | 0.67 |
|  | Vibration at 25 Hz | 1.94 | 1.49 | -1.07 | 4.94 | 1.30 | 0.20 |
|  | σ_u_ | 5.47 | | | | | |
|  | σ_є_ | 4.82 | | | | | |
|  |  |  |  |  | |  |  |
| **Semitendinosus** | Intercept | 22.51 | 1.54 | 19.37 | 25.65 | 14.57 | 0.00 |
|  | Non-vibration | -1.89 | 1.23 | -4.37 | 0.58 | -1.55 | 0.13 |
|  | Vibration at 25 Hz | 0.41 | 1.23 | -2.06 | 2.89 | 0.34 | 0.74 |
|  | σ_u_ | 5.86 | | | | | |
|  | σ_є_ | 3.97 | | | | | |
|  |  |  |  |  | |  |  |
| **Gluteus maximus** | Intercept | 11.47 | 1.01 | 9.40 | 13.53 | 11.37 | 0.00 |
|  | Non-vibration | -1.41 | 0.69 | -2.81 | -0.01 | -2.04 | 0.08 |
|  | Vibration at 25 Hz | 0.30 | 0.69 | -1.09 | 1.70 | 0.43 | 0.67 |
|  | σ_u_ | 4.04 | | | | | |
|  | σ_є_ | 2.23 | | | | | |
|  |  |  | | | | | |
| **Gastrocnemius medialis** | Intercept | 37.19 | 2.17 | 32.75 | 41.62 | 17.11 | 0.00 |
|  | Non-vibration | -0.87 | 1.55 | -4.00 | 2.26 | -0.56 | 0.58 |
|  | Vibration at 25 Hz | -0.18 | 1.55 | -3.31 | 2.94 | -0.12 | 0.91 |
|  | σ_u_ | 8.60 | | | | | |
|  | σ_є_ | 5.03 | | | | | |
|  |  |  | | | | | |
| **Gastrocnemius lateralis** | Intercept | 51.28 | 3.84 | 43.37 | 59.20 | 13.35 | 0.00 |
|  | Non-vibration | 0.27 | 1.94 | -3.65 | 4.18 | 0.14 | 0.89 |
|  | Vibration at 25 Hz | -0.41 | 1.94 | -4.32 | 3.50 | -0.21 | 0.83 |
|  | σ_u_ | 16.45 | | | | | |
|  | σ_є_ | 6.29 | | | | | |

**Supplementary Table 7.** Linear mixed model for suspended hamstring curl conditions (eccentric phase) with muscle activity as the dependent variable.

ES = coefficient estimate; SE = standard error; 95% CI = 95% confidence intervals; t = t- value; p = p-value; σ_u_ = standard deviation of participant; σ_є_ = standard deviation of residual. The “suspended hamstring curl with vibration at 40 Hz” was used as reference categories for this model in the exercise condition variable.
