# Supplementary material for: sEMG Activity in Superimposed Vibration on Suspended Supine Bridge and Hamstring Curl
Source: Front Physiol. 2021 Aug 11;12:712471. doi: 10.3389/fphys.2021.712471 (PMC8385437; doi:10.3389/fphys.2021.712471)
Supplement: Supplementary file 8 [file Table_8.DOCX]

| **Suspended hamstring curl: concentric phase** | | | | | | | |
| --- | --- | --- | --- | --- | --- | --- | --- |
|  | **Parameter** | **ES** | **SE** | **95%CI** | | **t** | **p** |
|  |  |  |  | Lower | Upper |  |  |
| **Global activity** | Intercept | 26.86 | 1.13 | 24.54 | 29.18 | 23.79 | 0.00 |
|  | Non-vibration | -1.41 | 0.66 | -2.75 | -0.09 | -2.16 | 0.70 |
|  | Vibration at 25 Hz | -0.28 | 0.66 | -1.60 | 1.04 | -0.43 | 0.67 |
|  | σ_u_ | 4.72 | | | | | |
|  | σ_є_ | 2.13 | | | | | |
| **Suspended hamstring curl: eccentric phase** | | | | | | | |
|  | **Parameter** | **ES** | **SE** | **95%CI** | | **t** | **p** |
|  |  |  |  | Lower | Upper |  |  |
| **Global activity** | Intercept | 24.48 | 1.04 | 22.34 | 26.63 | 23.47 | 0.00 |
|  | Non-vibration | -0.83 | 0.60 | -2.05 | 0.39 | -1.37 | 0.18 |
|  | Vibration at 25 Hz | 0.29 | 0.60 | -0.92 | 1.51 | 0.49 | 0.63 |
|  | σ_u_ | 4.36 | | | | | |
|  | σ_є_ | 1.96 | | | | | |
|  |  |  | | | | | |

**Supplementary Table 8.** Linear mixed model for suspended hamstring curl conditions (concentric and eccentric phase) with global activity as the dependent variable.

ES = coefficient estimate; SE = standard error; 95% CI = 95% confidence intervals; t = t- value; p = p-value; σ_u_ = standard deviation of participant; σ_є_ = standard deviation of residual. The “suspended hamstring curl with vibration at 40 Hz” was used as reference categories for this model in the exercise condition variable.
