# Supplementary material for: sEMG Activity in Superimposed Vibration on Suspended Supine Bridge and Hamstring Curl
Source: Front Physiol. 2021 Aug 11;12:712471. doi: 10.3389/fphys.2021.712471 (PMC8385437; doi:10.3389/fphys.2021.712471)
Supplement: Supplementary file 9 [file Table_9.DOCX]

|  | **OMNI-Res** | | | | |
| --- | --- | --- | --- | --- | --- |
|  | **Non-vibro vs vibro 25** |  | **Non-vibro vs vibro 40** |  | **Vibro 25 vs vibro 40** |
|  | **%** |  | **%** |  | **%** |
| Suspended supine bridge | 17.98 |  | 32.43 |  | 12.24 |
| Suspended hamstring curl | 9.82 |  | 10.71 |  | 0.81 |

**Supplementary Table 9.** Percentage of change for the OMNI-Res under suspended supine bridge and suspended hamstring curl conditions.
